# Utilization of Field Enhancement in Plasmonic Waveguides for Subwavelength Light-Guiding, Polarization Handling, Heating, and Optical Sensing

**DOI:** 10.3390/ma8105341

**Published:** 2015-10-09

**Authors:** Daoxin Dai, Hao Wu, Wei Zhang

**Affiliations:** 1 Centre for Optical and Electromagnetic Research, State Key Laboratory for Modern Optical Instrumentation, East Building No. 5, Zijingang Campus, Zhejiang University, Hangzhou 310058, China; leowu@zju.edu.cn; 2 Chongqing Institute of Green and Intelligent Technology, Chinese Academy of Sciences, Chongqing 400714, China

**Keywords:** plasmonic nanostructures, waveguide, polarization-handling, heating, sensing, silicon hybrid plasmonics

## Abstract

Plasmonic nanostructures have attracted intensive attention for many applications in recent years because of the field enhancement at the metal/dielectric interface. First, this strong field enhancement makes it possible to break the diffraction limit and enable subwavelength optical waveguiding, which is desired for nanophotonic integrated circuits with ultra-high integration density. Second, the field enhancement in plasmonic nanostructures occurs only for the polarization mode whose electric field is perpendicular to the metal/dielectric interface, and thus the strong birefringence is beneficial for realizing ultra-small polarization-sensitive/selective devices, including polarization beam splitters, and polarizers. Third, plasmonic nanostructures provide an excellent platform of merging electronics and photonics for some applications, e.g., thermal tuning, photo-thermal detection, *etc.* Finally, the field enhancement at the metal/dielectric interface helps a lot to realize optical sensors with high sensitivity when introducing plasmonic nanostrutures. In this paper, we give a review for recent progresses on the utilization of field enhancement in plasmonic nanostructures for these applications, e.g., waveguiding, polarization handling, heating, as well as optical sensing.

## 1. Introduction

Surface-plasmon polaritons (SPPs) are well known as a kind of electromagnetic waves coupled to the collective oscillations of free electrons, and can be supported at the metal/dielectric interface [1,2]. Particularly, the plasmonic waveguides consisting of metal/dielectric interfaces support SPP modes with field enhancement and light localization at the metal/dielectric interfaces. In order to have a low loss for the SPP mode, the complex dielectric function ε_m_ of metal should satisfy the following two conditions [2]: (1) ε_m_re_ < 0; (2) ε_m_im_ << ε_m_re_, where ε_m_re_ and ε_m_im_ are the real part and the imaginary part of the metal’s dielectric function ε_m_. As one of the most important properties for SPPs, the field enhancement at the metal surface makes plasmonic waveguides very useful for many applications of subwavelength light-guiding polarization-handling, heating and detecting, as well as optical sensing, *etc*.

The field enhancement at the metal/dielectric interface makes it possible to break the diffraction limit and realize subwavelength optical waveguides, which has been desired for a long time in order to satisfy the increasing demands for ultra-dense photonic integrated circuits (PICs) [3]. People have proposed and developed various SPP waveguides, including metal nano-slot waveguides [4,5,6,7] and metal V-groove waveguides [8]. However, these nanoplasmonic waveguides have large loss from the metal absorption, and thus the propagation distance is limited at the order of several microns. More recently hybrid plasmonic waveguides [9,10,11,12,13,14] have been proposed as an excellent candidate for simultaneously achieving a nano-scale light confinement as well as relatively long propagation distance by including a low-index nano-slot between a high-index region and a metal region. SOI (silicon-on-insulator)-compatible hybrid plasmonic waveguide is even more attractive because the intrinsic advantages of silicon photonics is integrated [12]. It is also convenient to have an efficient coupling between the silicon hybrid plasmonic waveguide and SOI nanowires by utilizing the butt-coupling [15] or the evanescent coupling approaches [16,17], so that these two types of nano-waveguides can work together seamlessly. There are lots of works on silicon hybrid plasmonic waveguides and devices developed since 2009 [12], which will be reviewed in Section 2.

Since SPP effect occurs for the polarization mode whose electrical field is perpendicular to the metal-dielectric interface only, the mode behavior in SPP nanostructures usually have very strong polarization dependence. For the guided SPP modes, not only the effective index (including the real part as well as the imaginary part) but also the field profiles are strongly polarization-sensitive. This is not good when one tries to realize polarization-insensitive PICs. However, on the other hand, it is very helpful for designing ultrasmall polarization-handling devices, including polarizers [18,19,20,21,22,23,24], polarization-beam splitters (PBS) [25,26,27,28,29,30,31], as well as polarization rotators [32,33,34,35,36], which are very useful for various applications with polarization handling [37]. This will be reviewed in Section 3. 

SPP waveguides having metal strips offer a platform of merging electronics and photonics on a single chip [3], which is promising to realize optoelectronic integrated circuits (OEICs) for efficient signal generation, modulation, and detection. For example, a metal strip is often used as a micro-heater for thermally-tuning photonic integrated devices [38,39]. Particularly, for silicon hybrid plasmonic waveguides, the metal cap on the top of the silicon core can intrinsically serve as a nano-heater to heat the silicon core very efficiently because the insulator layer between the metal strip and the silicon core is very thin. In this case, the temperature at the silicon core region is almost as high as that at the metal-strip heater, which is good to achieve a large thermal tuning. It is also helpful for the improvement of the temporal response. Thus it becomes possible to realize fast thermal tuning with a large range. Furthermore, the ultrasmall volume to be heated in a silicon hybrid plasmonic waveguide is reduced greatly so that very high energy-efficiency is obtainable. Another possible application merging electronics and photonics with SPP waveguides might be photodetection by utilizing the thermal-resistance effect of metal (heated by the incident optical power). An electrical Wheatstone bridge can be applied to accurately measure the resistance so that the resistance change of the metal strip has very high sensitivity. Particularly, for silicon hybrid plasmonic waveguides, the nano-scale confinement of light enhances the thermal-resistance effect due to the metal absorption so that improved performances can be achieved in comparison with the traditional long-range SPP waveguides. A summary for the electronics-photonics mergence of plasmonic nanostructures will be given in Section 4.

Finally, the field enhancement at the metal/dielectric interface also helps a lot to improve the interaction between the evanescent field and the medium contacted with the metal layer. In this case, the absorption and the propagation constant of the SPP mode are very sensitive to the change of the refractive index for the medium. As a result, it is promising to realize optical sensors with high sensitivity by using plasmonic nanostrutures. Particularly, the surface plasmon resonance (SPR) effect has been used very widely for bio- or chemo-sensing owing to its high sensitivity. For example, the concentration of special ions can be traced by monitoring the localized SPR peak wavelength [40] and the SPR chip can be used to detect and recognize specific biomolecules [41], which will be reviewed briefly in Section 5.

In this paper, a review will be given on recent progress on the utilization of field enhancement in SPP nanostructures for the applications of subwavelength waveguiding, strong polarization-handling, efficient heating and detection, as well as high-sensitivity optical sensing, *etc*.

## 2. Subwavelength Waveguides and Devices Based on Plasmonic Nanostructures

**Figure 1:**
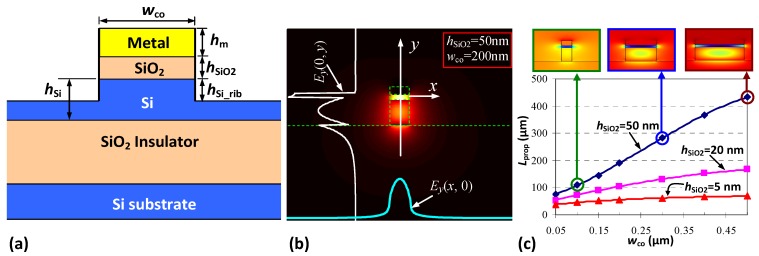
(**a**) The cross section of the present hybrid plasmonic waveguide with a metal cap on a silicon-on-insulator rib; (**b**) The calculated field distribution for the major component *E_y_*(*x*, *y*) of the quasi-TM fundamental mode of the present hybrid plasmonic waveguide; (**c**) The dependence of propagation distance *L*_prop_ on the core with *w*_co_, when the thicknesses of SiO_2_ are *h*_SiO2_ = 5, 20, 50 nm respectively (insets: the field profiles) [12].

Breaking the diffraction limit to realize subwavelength waveguides and devices have been attracting intensive attention for many years in the field of integrated photonics. SPP waveguides based on metal nanostructures were regarded as one of the most effective platform for subwavelength waveguiding and great progress has been achieved in the past decades. Particularly, the recently developed hybrid plasmonic waveguides have been considered as one of the most attractive options because of the possibility to achieve low-loss subwavelength waveguiding [42,43,44,45,46,47,48,49,50,51,52,53,54,55,56,57,58,59,60,61,62,63,64,65,66,67,68,69,70,71,72,73,74,75,76,77,78]. Among various hybrid plasmonic waveguides, an SOI-compatible hybrid nanoplasmonic waveguide, proposed for the first time by Dai *et al.* [12], is one of the most attractive designs as silicon photonics is CMOS (complementary metal oxide semiconductor) compatible and has become very popular for realizing PICs. The silicon hybrid nanoplasmonic waveguide proposed in [12] consists of an SOI nanowire with a metal cap atop, as shown in Figure 1a. Here the silicon layer can be etched deeply or shallowly. According to the boundary condition for the normal component of the electrical field, a hybrid plasmonic mode is supported with a field enhancement at the thin low-index region for the transverse magnetic (TM) polarization, as shown in Figure 1b.

It can be seen that the surface plasmonic effect in this structure occurs at the metal bottom-surface, which is as smooth as the top surface of the low-index thin film. Thus it is helpful to achieve low scattering loss of surface in comparison with those hybrid plasmonic waveguide utilizing the metal top-surface (which is quite rough usually due to the deposition process). Many novel hybrid nanoplasmonic waveguides with some modifications have been proposed and demonstrated experimentally in the following years [42,43,44,45,46,47,48,49,50,51,52,53,54,55,56,57,58,59,60,61,62,63,64,65,66,67,68,69,70,71,72,73,74,75,76,77,78]. For example, a modified hybrid plasmonic waveguide with a metal plate on the top was demonstrated in order to obtain a good adherence between the metal film and the low-index nano-slot layer [42,43]. An extremely small mode area can be achieved with another modified hybrid plasmonic waveguide with an inverted metal wedge cap [44,45]. It is also possible to have TE-type silicon hybrid nanoplasmonic waveguides (working for TE polarization) by introducing e.g., double low-index vertical nano-slots at both sides of a high-index region [46,47,48,49,50,51].

Figure 1c illustrates the dependence of the propagation distance on the core width when choosing different SiO_2_ nano-slot thickness *h*_SiO2_. It can be seen that the mode confinement and the propagation distance can be easily modified by choosing the structural parameters appropriately. For the present silicon hybrid nanoplasmonic waveguide, the mode area can be as small as ~50 × 5 nm^2^ and the propagation distance can be as long as 10^2^~10^3^ μm [12], which is attractive for realizing ultra-dense PICs. Furthermore, for SOI-compatible hybrid nanoplasmonic waveguides and devices, the fabrication is similar to the regular silicon photonic integrated circuits. Thus, it is convenient to integrate a nanoplasmonic integrated circuit and a nanophotonic integrated circuit on the same silicon chip monolithically.

**Figure 2:**
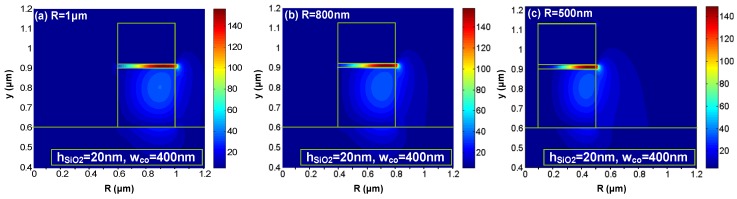
Electrical field distribution *E_y_*(*x*, *y*) of the TM fundamental mode for the cases of (**a**) *R* = 1 μm; (**b**) *R* = 800 nm; (**c**) *R* = 500 nm. The other parameters are: the Si core thickness *h*_Si_ = *h*_Si_rib_ = 300 nm, the SiO_2_ nano-slot thickness *h*_SiO2_ = 20 nm, the core width *w*_co_ = 400 nm [16].

**Figure 3:**
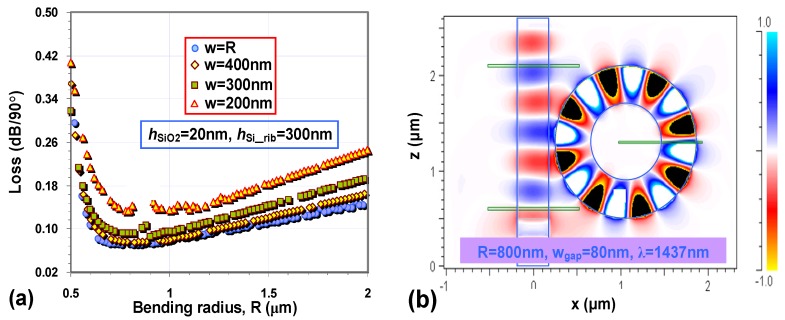
(**a**) The calculated bending loss for bent hybrid plasmonic waveguides (at 1550 nm); (**b**) The electrical field distribution *E_y_*(*x*, *z*) in a submicron-donut resonator with *R* = 800 nm, and *w*_g_ = 80 nm when it is on-resonance. The other parameters are: the Si core thickness *h*_Si_ = *h*_Si_rib_ = 300 nm, the SiO_2_ nano-slot thickness *h*_SiO2_ = 20 nm [16].

Silicon hybrid nanoplasmonic waveguides not only provide the ability for very tight optical confinement (field enhancement/localization) but also enable ultra-sharp bending, which is a key in determining the integration density of the functionality elements, such as power splitters, sub-micron cavities, *etc*. Figure 2a–c show the electrical field distribution *E_y_*(*x*, *y*) of the TM fundamental mode for the cases of *R* = 1 μm, 800 nm, and 500 nm, respectively [16]. From these figures, it can be seen that there is a significant field enhancement in the low-index slot region and the light is still very well confined in the slot region even when the bending radius *R* is as small as 500 nm (~λ/3, λ = 1550 nm). The peak of the electrical field shifts outward as expected when the radius decreases. One should note that there is an optimal bending radius for minimizing the total propagation loss in a 90°-bending, as shown in Figure 3a. The reason is that a hybrid nanoplasmonic waveguide has some intrinsic loss due to the metal absorption, which is proportional to the propagation distance *L* = *R*π/2. Such hybrid nanoplasmonic waveguides can be then utilized to realize a submicron resonator. For example, a submicro-donut resonator with a bending radius *R* of 800 nm and a moderate Q-value was designed and characterized (see Figure 3b [16]).

**Figure 4:**
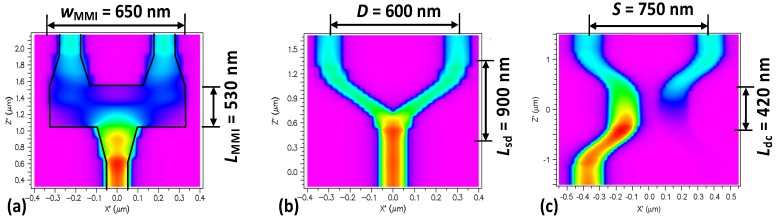
The designed 3 dB power splitters based on (**a**) a 1 × 2 MMI coupler with the length *L*_MMI_ = 530 nm and the width *W*_MMI_ = 650 nm; (**b**) a Y-branch with the length *L*_S-bend_ = 900 nm and the separation *D* = 600 nm; and (**c**) a direction coupler with the length *L*_dc_ = 420 nm and the separation *D* = 750 nm. The waveguide width *w*_co_ = 150 nm, the thickness of the SiO_2_ slot layer, *h*_SiO2_ = 20 nm [79].

The hybrid nanoplasmonic waveguides can also enable sub-μm^2^ power splitters [79,80] as shown in Figure 4a–c. From these figures, it can be seen that these sub-μm^2^ power splitters, including 1 × 2 MMI (multimode interference) couplers, Y-branches and direction couplers (DCs) have ultrasmall footprints as well as high power transmissions of ~90% [79]. These fundamental blocks will be useful for establishing ultra-dense photonic integrated circuits in the future.

**Figure 5:**
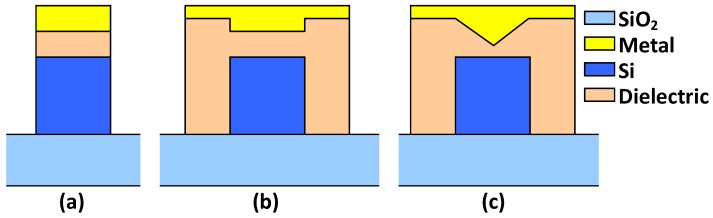
Cross sections of silicon hybrid plasmonic waveguides with a standard metal cap [12] (**a**); an inverted metal rib cap [42,43] (**b**); and an inverted metal wedge cap [44,45] (**c**), respectively. [86]

Silicon hybrid plasmonic waveguides also provide an excellent platform of nonlinear PICs for the applications of all-optical signal processing in comparison with SOI nanowires [81,82] and silicon nano-slot waveguides [82,83] because TM polarization mode is well confined in the narrow slot between the metal region and the high-index layer, leading to a strong enhancement in the power density. Furthermore, the low-index slot region can be filled with nonlinear optical materials, contributing to improved nonlinear photonic effects. Furthermore, as the power density in the silicon core region reduces, the two-photon absorption (TPA) and the TPA-generated free carrier effects (FCE) become less and higher power is allowed for achieving stronger nonlinear photonic effects. In [84,85,86], the enhanced nonlinear photonic effects in silicon hybrid plasmonic waveguides have been well investigated. It is shown that the nonlinear effects in hybrid plasmonic waveguides is highly dependent on the waveguide loss, which is different from the pure-dielectric optical waveguides (e.g., SOI nanowires). There are also several types of structures available (shown in Figure 5) for silicon hybrid plasmonic waveguides with different localized power densities. According to the comparison given by Pitilakis *et al.* in [86], one can obtain an exceptionally high nonlinear parameter γ > 104 m^−1^·W^−1^ combined with an FCE power threshold larger than 1 W (even in CW) by using the silicon hybrid plasmonic waveguides with an inverted metal wedge cap, in which the optical field is localized in a nanosized nonlinear polymer gap formed between a metal wedge and an underlying silicon wire.

As a brief summary for this section, plasmonic waveguides have been well recognized and popular due to the ability of breaking the diffraction limit and realizing subwavelength light-guiding. As one of the most attractive plasmonic waveguides, the SOI-compatible hybrid nanoplasmonic waveguide has been developed very well. The challenge is that it is still difficult to realize large-scale PICs based on hybrid plasmonic waveguides due to the loss issue even though the intrinsic loss of a hybrid plasmonic waveguide is relatively low. A possible solution is introducing some gain medium to compensate the intrinsic loss of plasmonic waveguides [43,87,88]. For silicon hybrid plasmonics, the gain medium could be realized by using Er-doping, quantum dots, Si nano-crystals, *etc*. When net gain is achieved, it becomes possible to realize a deep subwavelenth plasmonic laser [88]. Another strategy is combining silicon hybrid plasmonic waveguides and low-loss SOI nanowires together so that silicon hybrid plasmonic waveguides are used locally. In this case, it is important to have efficient coupling between the silicon hybrid plasmonic waveguide and SOI nanowires, which can be realized by utilizing the butt-coupling [15] or the evanescent coupling approaches [16,17]. When using the butt-coupling method, the coupling efficiency can be as high as 70%~80% even with a very short simple mode converter [15]. The evanescent coupling efficiency could be as high as 100% almost when the two coupled waveguides satisfy the phase-matching condition [17].

## 3. Polarization-Handling Devices Based on Plasmonic Nanostructures

For silicon hybrid plasmonic waveguides, not only the modal effective index (including the real part as well as the imaginary part) but also the modal field profiles are strongly polarization-sensitive. From the comparison between the polarization dependences of the silicon hybrid plasmonic waveguide and an SOI nanowire [89], it can be seen that these two types of waveguides have similar field profiles for TE polarization mode because the plasmonic effect exists for TM polarization only. In contrast, the field profiles for their TM polarization modes are very different due to the surface plasmonic effect. As mentioned before, the TM modal field for a silicon hybrid plasmonic waveguide has an enhancement in the nano-slot region. Similarly, the two types of optical waveguides have very different effective indices for TM polarization while the effective indices for TE polarization are not different greatly. This opens a door to realize excellent ultrasmall polarization-handling devices, including polarizers [18,19,20,21,22,23,24], PBSs [25,26,27,28,29,30] as well as polarization rotators [32,33,34,35], which is very useful for many applications. In this paper, we focus on the polarizers and PBSs based on silicon hybrid plasmonic waveguides.

### 3.1. Ultrasmall PBSs Based on Hybrid Nanoplasmonic Waveguide Structures

On-chip PBSs have been realized by utilizing various structures, e.g., multimode interference (MMI) structures [90], directional couplers (DCs) [91,92,93,94], Mach-Zehnder interferometers (MZIs) [95,96], arrayed-waveguide grating [97], photonic-crystal structures [98], MMI couplers [99,100], and grating structures [101]. Recently, silicon hybrid nanoplasmonic waveguides have been paid lots of attention for realizing ultrasmall PBSs with asymmetric DCs (ADCs) [25,26,27,28,29] and special MMI structures [30], which will be summarized in the following part.

#### 3.1.1. PBSs Based on ADCs

Asymmetric direction coupling systems have been proved to be an excellent option for the realization of ultra-small and broadband PBSs [102,103]. In order to make an asymmetric direction coupling system, a simple way is choosing two types of optical waveguides (whose birefringences are different) to form the coupling region [104]. In this way, the coupling region can be designed to satisfy the phase-matching condition for only one polarization, which is the key to separate two polarization modes efficiently.

**Figure 6:**
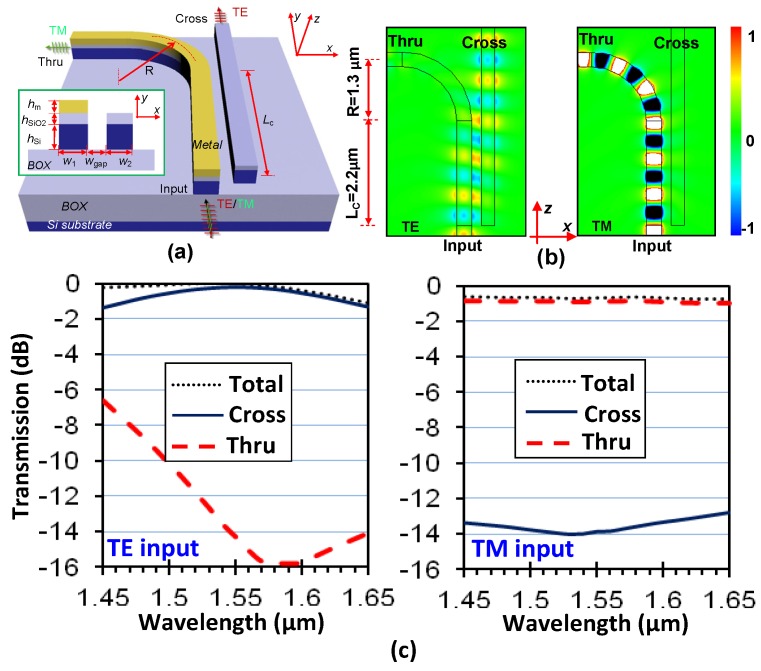
(**a**) Configuration of the ultrasmall PBS based on an ADC with a silicon hybrid nanoplasmonic waveguide; (**b**) Simulated light propagation for TE- and TM-polarizations; (**c**) Simulated transmission responses for TE- and TM-polarizations. The parameters are: *L*_c_ = 2.2 μm, *R* = 1.3 μm, *w*_1_ = 310 nm, *w*_2_ = 280 nm, *w*_gap_ = 200 nm, *h*_Si_ = 230 nm, *h*_SiO2_ = 50 nm and *h*_m_ = 100 nm [28].

Figure 6a shows an ADC consisting of a silicon hybrid nanoplasmonic waveguide and a SOI nanowire, which was proposed to work as an ultracompact and broadband PBS [28]. In such a coupling system, the two waveguides were designed to be phase-matched for their TE polarization modes (which have similar modal fields as well as the effective indices) so that the input TE polarization can be cross-coupled completely after propagating an optimal distance. In contrast, there is a significant phase mismatching between the TM polarization modes for the two waveguides, and thus the evanescent coupling between them becomes very weak. The simulation results given in [28] shows that the optimal length for the coupling region is as short as 2.2 μm even when the width of the gap between two waveguides is chosen as large as 200 nm (which is large enough for easy fabrication). The simulated light propagation of the designed PBS is shown in Figure 6b, from which one can see the two polarizations are separated efficiently as expected. Because the phase mismatching for TM polarization is significant over a broad band, it can be seen that the extinction ratio for TM polarization is high in the wavelength range from 1.45 μm to 1.65 μm. In contrast, for TE polarization, the wavelength response is very similar to a regular directional coupler, which limits the bandwidth of the PBS. Fortunately, the bandwidth is still more than 100 nm for an extinction ratio of 10 dB, and the designed PBS also has low excess loss of ~0.025 dB and ~0.66 dB for TE and TM polarizations, respectively (see Figure 6c).

**Figure 7:**
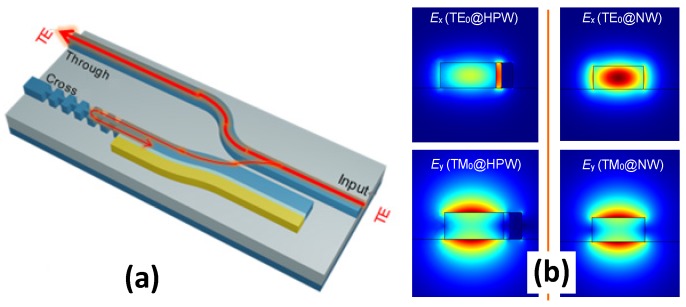

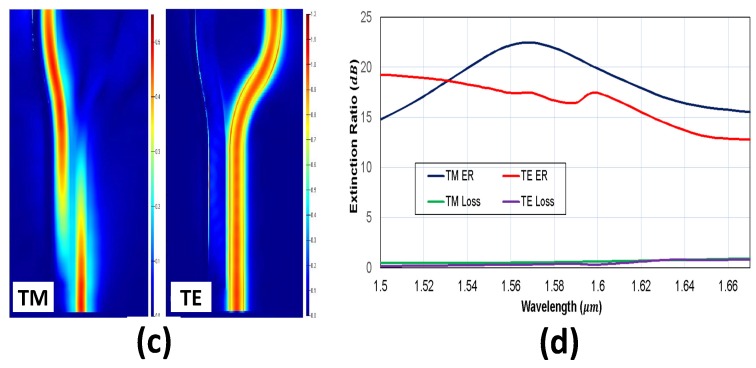
(**a**) Configuration of the modified ultrasmall PBS based on an ADC with a silicon hybrid nanoplasmonic waveguide; (**b**) The modal field profiles in a silicon hybrid plasmonic waveguide and an SOI nanowire; (**c**) Light propagation for TM and TM polarizations in the designed PBS; (**d**) The calculated wavelength-dependence of the designed PBS [105].

One should note that there are some additional mode conversion losses at the input port and the through port when this type of PBS is to be connected with regular SOI nanowires (which is low-loss for long-distance optical interconnects). In order to solve this problem, a modified PBS was proposed in [105], as shown in Figure 7a. The modified design consists of a *horizontal* hybrid nanoplasmonic waveguide and a SOI nanowire. The field distributions for both TE- and TM-polarization modes in these two types of waveguides are also shown in Figure 7b. It can be seen that their TM polarization modes are similar and thus the phase-matching condition can be satisfied possibly. Meanwhile, their TE polarization modes are very different because the TE polarization mode of the horizontal hybrid nanoplasmonic waveguide has a field enhancement in the nanoslot. For this PBS, the input port and the through port can be connected with a long SOI-nanowire for global interconnections without any additional mode converter. Consequently, there is no additional mode conversion loss. At the cross ports, there is some mode conversion loss when the silicon hybrid plasmonic waveguide is connected with an SOI nanowire (for e.g., long-distance interconnects). Fortunately, this mode conversion loss is very low because the TM polarization modes for these two waveguides are very similar (see Figure 7b). The simulated light propagation of the designed PBS is shown in Figure 7c, from which it can be seen that the two polarization modes are separated very well. The extinction ratio at the cross port can be improved further with a cascaded TM-passed polarizer [106], which reflects the residual TE polarization mode while the excess loss for the passed TM polarization mode is very low. With this design, the PBS has an ultra-compact footprint of ~2.9 μm × 5.8 μm, and achieves an extinction ratio >15 dB and a small excess loss <0.7 dB in the wavelength range from 1500 nm to 1620 nm.

#### 3.1.2. The PBS Based on an MMI Coupler

An MMI structure is very popular for photonic integrated circuits because of the easy fabrication. However, the polarization-dependence of an MMI structure is usually very weak [99], which means that it takes a very long length to separate the TE and TM modes. A shortened MMI-based PBS can be achieved by utilizing the so-called quasi imaging in an MMI section, however, the length of the device is still very long (e.g., *L*_MMI_ = ~1034 μm [100]).

**Figure 8:**
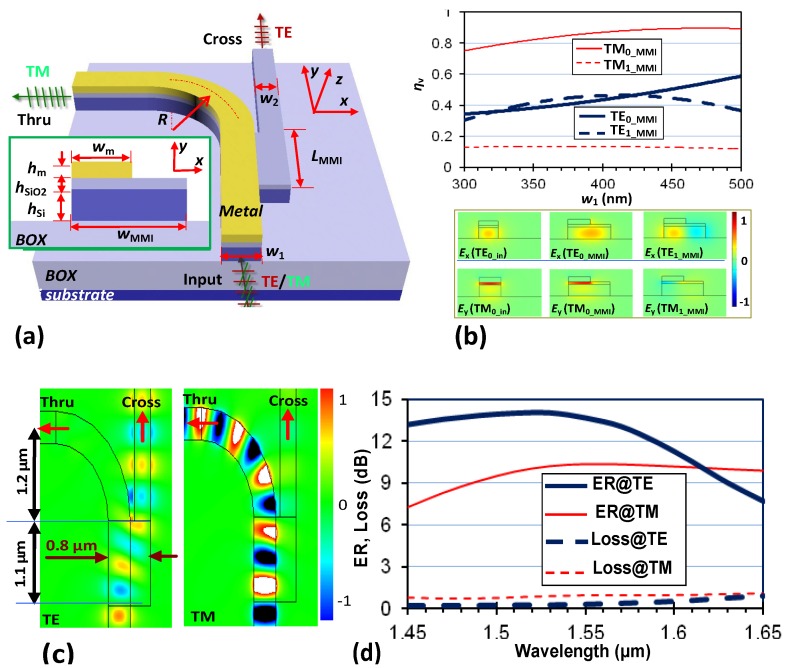
(**a**) Configuration of the PBS based on an MMI coupler with a silicon hybrid nanoplasmonic waveguide; (**b**) The mode excitation ratio η*_v_* for the modes in the MMI section; (**c**) the simulated light propagation in the designed PBS; (**d**) the calculated transmission responses. The parameters are: the Si core layer thickness *h*_Si_ = 230 nm, the SiO_2_ nano-slot thickness *h*_SiO2_ = 50 nm, the metal (Ag) thickness *h*_m_ = 100 nm, *w*_1_ = 420 nm, *w*_2_ = 300 nm, *w*_MMI_ = 800 nm, *R* = 1.2 μm [30].

In Reference [30], we proposed a novel extremely small PBS by using a special MMI structure as shown in Figure 8a. The novel MMI structure is partially covered by a metal strip to form a hybrid nanoplasmonic waveguide. In this design, the metal strips on the input waveguide and the MMI section have the same width. Thus the TM_0_ mode in the input section is very similar to that in the MMI section due to the hybrid nanoplasmonic effect (see Figure 8b). As a result, the TM_0_ mode in the MMI section is excited dominantly and the MMI effect does not happen almost when the TM polarization mode is launched from the input section. In this case, for TM polarization, light outputs from the through port dominantly. For TE polarization, the metal on the top influences the light propagation very slightly because there is no hybrid nanoplasmonic effect (see Figure 8b). As a result, both the fundamental and higher-order modes are excited in the MMI section and interfere happens between these two modes. A mirror image for TE polarization can be formed at the cross port when the MMI length is chosen optimally. As an example demonstrated in [30], the parameters are chosen as follows: λ = 1550 nm, *w*_1_ = 420 nm, *w*_2_ = 300 nm, *w*_MMI_ = 800 nm, *R* = 1.2 μm, and the MMI section *L*_MMI_ = 1.1 μm. The simulated light propagation in the designed PBS is shown in Figure 8c, and it is clearly seen that two polarizations are separated efficiently in such a short region. The designed PBS achieves an extinction ratio of >10 dB over a ~80 nm bandwidth, with excess losses as low as 0.32 dB and 0.88 dB for TE and TM polarizations respectively (see Figure 8d).

### 3.2. Polarizers

A polarizer is a basic device for polarization-handling and is used to improve the extinction ratio of polarized light. Regarding that the modal fields for TE- and TM-polarizations of silicon hybrid nanoplasmonic waveguides are very different, which indicates that the TE and TM polarization modes might be modulated very differently by some special lateral and vertical structures. In this way, TE-pass or TM-pass polarizers based on hybrid nanoplasmonic waveguides have been reported [18,19,20,21,22,23]. Among them, the grating structures based on silicon hybrid plasmonic waveguides have been proved to be an efficient way to realize the ultracompact TE-pass and TM-pass polarizers [22,23].

**Figure 9:**
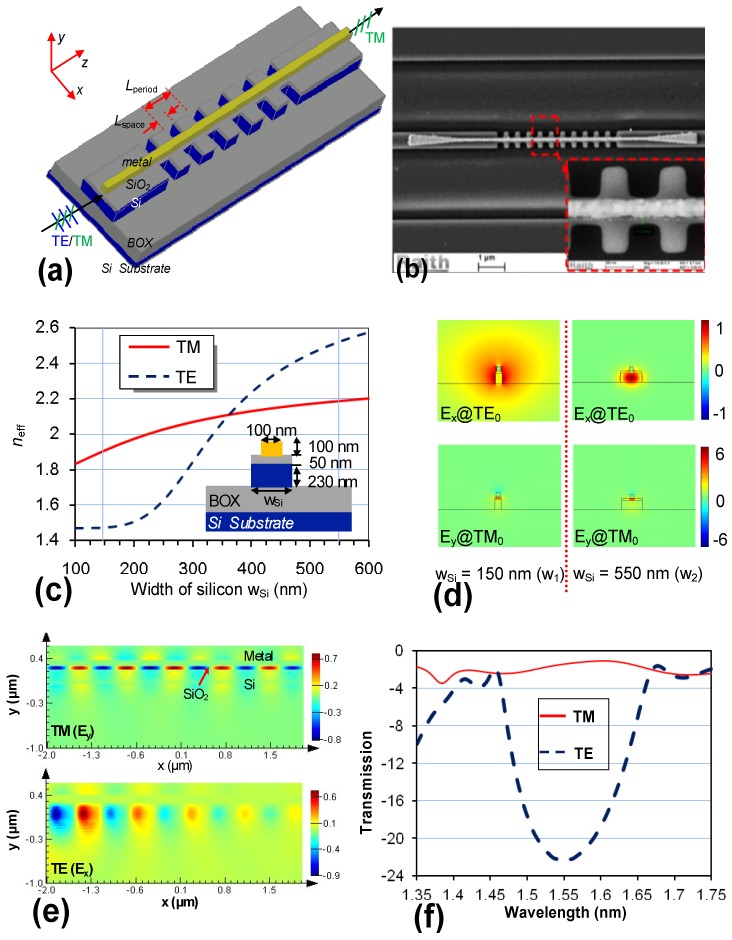
(**a**) Configuration of the TM-pass polarizer based on a silicon grating with a narrow metal strip on the top; (**b**) SEM picture for the fabricated polarizer; (**c**) the effective indices for TE- and TM-polarizations as the silicon core width *w*_Si_ varies from 100 nm to 600 nm; (**d**) the modal field profiles (*E_x_*, *E_y_*) for TE- and TM-polarizations when *w*_Si_ = 100 nm, and 500 nm, respectively; (**e**) Simulated light propagation in the designed polarizer for TE- and TM-polarizations; (**f**) Simulated transmission spectral responses [22].

In Reference [22], we proposed a novel TM-pass polarizer composed of a waveguide grating covered by a narrow metal strip in the middle, as shown in Figure 9a,b. According the electric field profiles of TE-polarization mode (see Figure 9c), one sees that TE polarization can be modulated greatly by the periodic variation in the waveguide width. As a consequence, the input TE polarization is reflected by the waveguide grating efficiently in the wavelength band around the Bragg wavelength. In contrast, TM polarization is influenced very little because it is very well confined in the region defined by the narrow metal strip. The TM-polarized light is “bounded” to propagate along the metal strip with a low scattering and reflection loss. Figure 9d,e shows the simulated light propagation in the designed structure with the following parameters: metal width *w_m_* = 100 nm, the period length *L*_period_ = 430 nm, and the period number *N* = 11. The corresponding length for this TM-passed polarizer is about 4.8 μm only and the corresponding transmission spectral responses are shown in Figure 9f. It can be seen that an extinction ratio of >20 dB is achieved over a bandwidth as large as ~50 nm.

**Figure 10:**
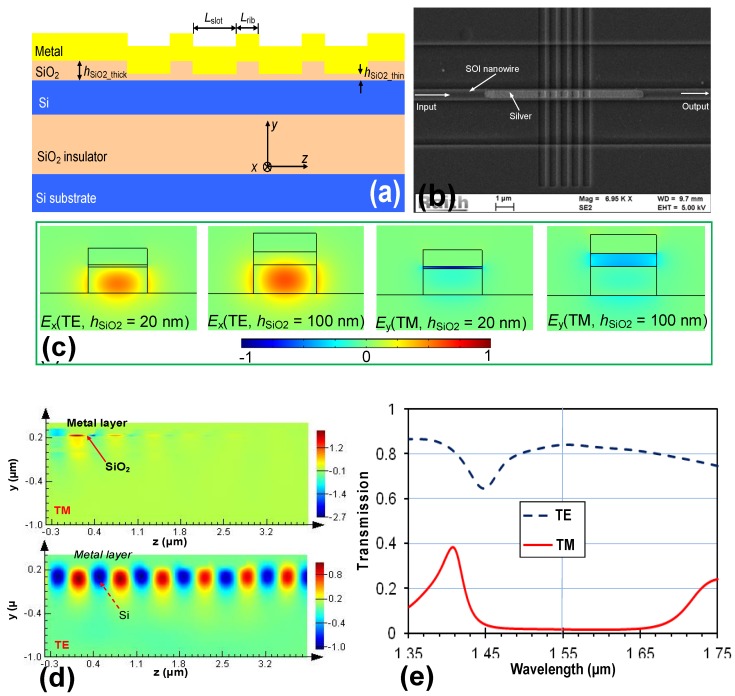
(**a**) Configuration of a TE-pass polarizer with a grating working for TM polarization; (**b**) SEM picture for the fabricated polarizer; (**c**) The modal field profiles when the SiO_2_-slot thickness *h*_SiO2_ are chosen differently (*h*_SiO2_ = 20 nm, and 100 nm, respectively); (**d**) Simulated light propagation in the designed polarizer; (**e**) Simulated transmission spectral responses for TE- and TM- polarization modes [23].

A TE-pass polarizer can also be realized by using a hybrid plasmonic waveguide grating designed for TM polarization. In Reference [23], the grating is formed by periodically modifying the thickness *h*_SiO2_ of the SiO_2_ layer, as shown in Figure 10a,b. Such a grating works for TM polarization very well because the propagation constant of TM polarization mode is very sensitive to the thickness of the SiO_2_ layer, while TE polarization is hardly affected by the modulated SiO_2_ thickness (see the mode profiles shown in Figure 10c). Therefore, when the operation wavelength is around the Bragg wavelength, the TM-polarized light will be reflected efficiently, as shown in Figure 10d. In this example, the following parameters are chosen for the grating structure according to the Bragg condition for TM_0_ mode: λ = 1550 nm, *N* = 5, *L*_slot_ = 400 nm, and *L*_rib_ = 200 nm. The corresponding length for the polarizer is ~3.1 μm only. From the calculated spectral responses shown in Figure 10e, it can be seen that the extinction ratio is about 18 dB and the excess loss is ~0.76 dB around the center wavelength. The bandwidth for achieving >15 dB extinction ratio is as wide as 200 nm.

As a summary for Section 3, it has been proven that plasmonic waveguides provide a good platform to realize on-chip polarization-handling devices with ultra-compact footprints. These reported polarization-handling devices have shown the potential of the seamless integration between SOI nanowires and silicon hybrid nanoplasmonics waveguides. In these examples, the silicon hybrid nanoplasmonics waveguides are used locally so that the excess losses for the realized polarization-handling devices are acceptably low. Therefore, it is predictable that the on-chip polarization-handling devices with plasmonic waveguides will be useful for the future ultra-dense nanophotonic integrated circuits.

## 4. Heating/Thermal Effects in Plasmonic Nanostructures

Devices utilizing thermal effects have been widely used today for tuning because of the simple design and easy fabrication. Silicon photonics is a promising candidate for the on-chip thermos-based devices due to its large thermal-optical coefficient and mature fabrication technology. Traditional silicon photonic devices with heating/thermal effects usually have a relatively large volume, which leads to bottlenecks in the performance and power consumption. The hybrid nanoplasmonic waveguide, with a very strong confinement of the optical field, provides a possible way to enhance the thermal-optical effect for functionality devices [107,108].

### 4.1. Energy-Efficient Thermal-Tuning for Silicon Hybrid Nanoplasmonic Devices

Thermal-tunable silicon photonic devices are very popular for realizing optical switches, variable optical attenuators. A traditional design is to put a metal strip as a micro-heater on the top of the silicon core with an upper-cladding insulator [39]. Particularly, the upper-cladding insulator should be thick enough to prevent notable absorption from metal micro-heater. In this case, the thermal flow from the micro-heater to the silicon core region is blocked seriously. Consequently, the temporal response becomes slow and the power consumption increases. As an alternative, the silicon plasmonic waveguide intrinsically have a metal strip atop, which can work as a nano-heater conveniently. Since the SiO_2_ insulator between the metal strip and the silicon core region is very thin, the heat transfer from the metal strip to the silicon core region becomes very efficient. Furthermore, a silicon hybrid plasmonic waveguide enables strong optical confinement so that the heating volume is reduced greatly, which makes it possible to realize low power-consumed thermal tuning.

**Figure 11:**
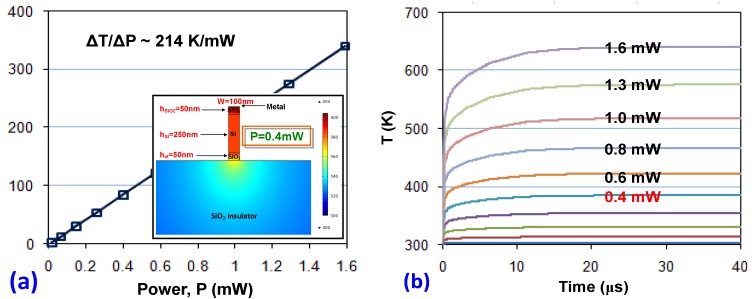
(**a**) The stable temperature variation as the power increases; inset: the simulated temperature profile when a 10 μm-long silicon hybrid plasmonic waveguide is heated by an power of 0.4 mW; (**b**) the temporal response when it is heated with different powers [107].

In Reference [107], the analysis of the thermal response for a heated silicon hybrid nanoplasmonic waveguide is presented. Figure 11a shows the simulated temperature profile in a silicon hybrid nanoplasmonic waveguide when heated with a power of 0.4 mW. In this example, the waveguide parameters are chosen as: the waveguide width *w =* 100 nm, the SiO_2_ thickness *h*_SiO2_ = 50 nm, the silicon-core thickness *h*_Si_ *=* 250 nm, the over-etched thickness *h*_et_ *=* 50 nm and the metal-strip length *L* = 10 μm. As expected, the temperature in the silicon core is very close to that in the metal strip because the SiO_2_ insulator layer is as thin as 50 nm. This indicates that high temperature in the silicon core region is achievable, which helps realize a large thermal-tuning range. From the calculation results shown in Figure 11a, it can be seen that the heating efficiency is as high as 214 K/mW and it is possible to achieve ~ 340 K with a power consumption of 1.6 mW. The calculated temporal responses are also shown in Figure 11b and the response time is about 500 ns (which is much faster than the traditional SOI nanowire heated with a micro-heater). 

### 4.2. Photo-Thermal Photodetector

Using photo-thermal effect provides a way to detect photons conveniently and cheaply. Particularly, the advantage of photo-thermal detectors is the ability to detect the long wave whose wavelength is beyond the working wavelength-range for traditional semiconductor photodetectors. Conventional plasmonic waveguides have been utilized to realize photodetectors with relatively low responsivity [109], limited working range [109], or slow response [109,110]. As discussed above, a hybrid nanoplasmonic waveguide has the ability to confine light in a very small volume, and thus the metal strip will be heated effectively when the input light is absorbed by the metal strip. In this case, there is a significant thermal-resistance effect to achieve a high responsivity and the resistance change can be measured easily electrically.

**Figure 12:**
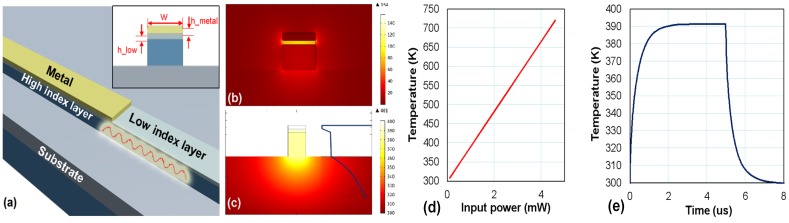
(**a**) Configuration of the photo-thermal detector based on a silicon hybrid plasmonic waveguide; (**b**) The optical field distribution of the silicon hybrid plasmonic waveguide with a core width *w* = 300 nm @ λ = 3.39 μm; (**c**) The temperature profile of the silicon hybrid plasmonic waveguide when 1 mW power is applied in the metal strip; (**d**) The maximal temperature in the metal strip as the input power varies; (**e**) The temporal response of the temperature of the metal strip with an applied power of 1 mW [108].

In Reference [108], a photo-thermal detector is proposed for the first time by using a hybrid nanoplasmonic waveguide, as shown in Figure 12a. In this design, the incident TM-polarized light guided by a silicon nanowire is incident to the hybrid plasmonic waveguide section and absorbed by the metal strip atop, which results in a temperature increase. Figure 12b,c show the calculated modal field profile (@ λ = 3.39 μm) and the temperature profile of the hybrid plasmonic waveguide with an input power of 1 mW when choosing the following parameters: the SiO_2_ thickness *h*_low_ *=* 20 nm, the metal thickness *h*_metal_ = 20 nm, the waveguide width *w* = 300 nm and the metal strip length *L* = 10 μm. Figure 12d shows the maximum of the temperature in the metal strip as the input power increases. With this optimized structure, the theoretical responsivity is as high as 91 K/mW. The temporal response shown in Figure 12e indicates that the rise time and the decay time for the present photodetector are as short as less than 1.0 μs.

As a brief summary, the application of utilizing the heating/thermal effects in plasmonic nanostructures is a good representative of merging electric circuits and photonic circuits on the same chip. Particularly, the thermal-tuning with silicon hybrid nanoplasmonic waveguides provides a way to realize energy-efficient tunable/switchable silicon photonic devices, which is very important for future large scale photonic integrated circuits. The photo-thermal photodetector based on silicon hybrid nanoplasmonic waveguides provide a convenient and low-cost approach for on-chip photodetection, which will be useful for the application of optical sensing, particularly for realizing labs-on-chip.

## 5. Optical Sensing Based on Plasmonic Nanostructures

It is well known that plasmonic nanostructures usually have strong field enhancement at the metal/dielectric interface, which helps realize optical sensors with high sensitivity. Particularly, the SPR effect has been widely used for bio- or chemo- sensing owing to its high sensitivity. For example, the concentration of special ions can be traced by monitoring the localized SPR peak wavelength [40]. SPR chips with special modified surfaces can be used to distinguish and detect the corresponding bio-molecules [41]. The SPR technique is also an efficient tool for the electrochemical process, by monitoring the response to potential perturbation [111].

**Figure 13:**
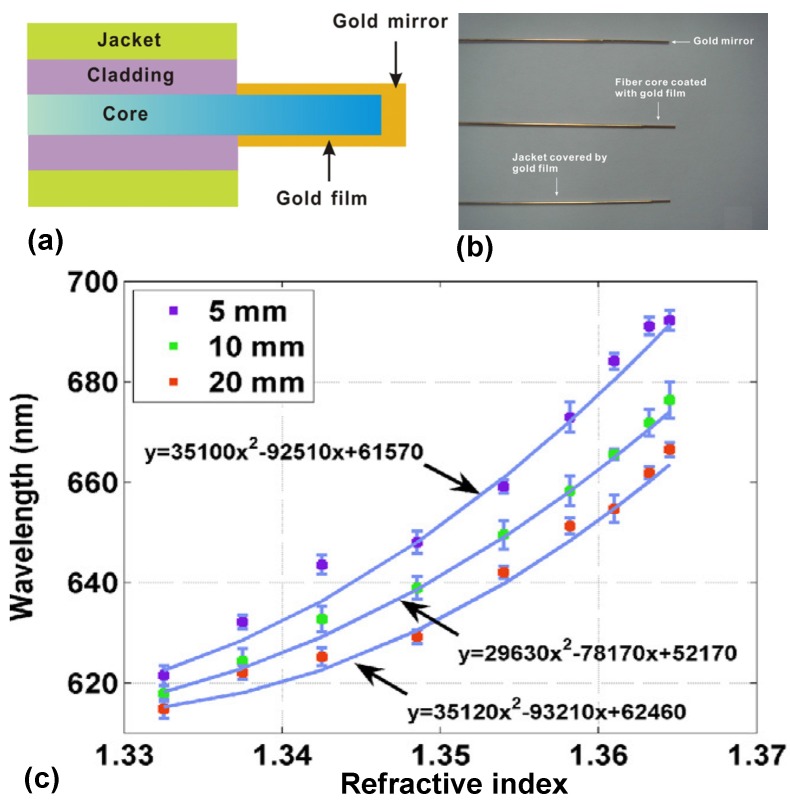
(**a**) Structure of a reflection type SPR fiber optic sensor; (**b**) Photo of SPR fiber optic sensors; (**c**) The resonance wavelength shift as the refractive index of the ethanol-water mixture increases in the range from 1.33 to 1.37 [112].

In Reference [112], a fiber optic sensor based on surface plasmon resonance was proposed to monitor the refractive index change of flowing solution. As shown in Figure 13a, the jacket and polymer-cladding of multimode fiber tips (with a core diameter of 600 μm and a numerical aperture of 0.37) are removed over an areas with a length of 5 mm, 10 mm and 20 mm for sensing. A 50 nm thick gold layer was then coated on the lateral side of the uncladded fiber, while a 100 nm thick gold layer is deposited on the adjacent end surface of the fiber to reflect the incident light. As the solution of different refractive index flow through the SPR probe, the reflected spectrums are captured. Figure 13c show the relationship between the resonance wavelength shift and the refractive index. The measured sensitivities to refractive index are 3186, 2613, and 2541 nm/RIU when the refractive index is around 1.3632 when the SPR probes have a sensing region as long as 5 mm, 10 mm, and 20 mm, respectively.

## 6. Conclusions

In this paper, we have given a review for recent progresses on the utilization of the field enhancement in plasmonic nanostructures for the applications of waveguiding, polarization handling, heating, as well as optical sensing. Among various plasmonic nanostructures for subwavelength waveguiding, a silicon hybrid plasmonic waveguide is one of the most promising options because of the CMOS compatibility, flexible design, relatively low loss, as well as very strong optical field confinement/localization. It has been proven that silicon hybrid plasmonic waveguides enable sub-micron bending as well as sub-μm^2^ photonic integrated devices (e.g., power splitters), which is desired for realizing ultra-dense photonic integrated circuits. Since plasmonic nanostructures usually have the very strong birefringence, ultra-small polarization-sensitive/selective devices can be realized. For example, the PBS based on an ADC consisting of a silicon hybrid plasmonic waveguide has a footprint as small as ~1.9 × 3.7 μm^2^. When using a special MMI structure partially covered by a metal strip, the PBS can be even smaller (~1.8 × 2.5 μm^2^). Ultra-shot polarizers can be also realized by utilizing a silicon plasmonic waveguide with the structure of gratings. The proposed and demonstrated TE-pass and TM-pass polarizers have a length of 3~5 μm. The metal layer in plasmonic nanostructures can be used as a part of an electrical circuit for some applications when it is desired for merging electronics and photonics. For example, it has been proposed to use the metal strip on the top of a silicon plasmonic waveguide as an energy-efficient nano-heater because the heating volume is reduced greatly and heat energy can be delivered very easily to the silicon core. It has also been demonstrated that the photo-thermal effect due to the absorption of metal in a silicon hybrid plasmonic waveguide can be utilized for light detection over a very broad wavelength range. This might be useful for an on-chip optical sensing system operating with a wavelength beyond the detection range of regular semiconductor photodiodes. Furthermore, such a kind of photodetector is cheap due to the easy design and fabrication. Finally, the utilization of plasmonic nanostructures for optical sensing has been discussed. Particularly, fiber-based plasmonic sensors is very promising because of the high sensitivity and the convenience for use in practice.

As a conclusion, plasmonic nanostructures with metals will play a very important role in photonic integrated circuits for subwavelength waveguiding, strong polarization-handling, efficient heating and detection, as well as high-sensitivity optical sensing. For the future development of plasmonic waveguides and devices, one should realize that it is still a challenge to achieve ultra-low intrinsic loss and ultra-high light confinement simultaneously. This issue might be solved partially by introducing some efficient gain medium with high gain coefficients. Another key to minimize the excess loss is that the metal-related fabrication processes should be improved so that high-quality metal thin film can be deposited in the defined area and smooth surfaces of metal can be achieved. More efforts and more experimental results are desired in the future.
